# Applicability of augmented reality in orthopedic surgery – A systematic review

**DOI:** 10.1186/s12891-020-3110-2

**Published:** 2020-02-15

**Authors:** Lukas Jud, Javad Fotouhi, Octavian Andronic, Alexander Aichmair, Greg Osgood, Nassir Navab, Mazda Farshad

**Affiliations:** 1 Department of Orthopedics, Balgrist University Hospital, University of Zurich, Forchstrasse 340, 8008 Zürich, Switzerland; 2 Computer Aided Medical Procedure, Johns Hopkins University, 3400 N Charles Street, Baltimore, 21210 USA; 3 Johns Hopkins Hospital, Department of Orthopedics Surgery, 1800 Orleans Street, Baltimore, 21287 USA; 4 Computer Aided Medical Procedure, Technical University of Munich, Boltzmannstrasse 3, 85748 Munich, Germany

**Keywords:** Augmented reality, Orthopaedic surgery, Trauma surgery, Image overlay

## Abstract

**Background:**

Computer-assisted solutions are changing surgical practice continuously. One of the most disruptive technologies among the computer-integrated surgical techniques is Augmented Reality (AR). While Augmented Reality is increasingly used in several medical specialties, its potential benefit in orthopedic surgery is not yet clear. The purpose of this article is to provide a systematic review of the current state of knowledge and the applicability of AR in orthopedic surgery.

**Methods:**

A systematic review of the current literature was performed to find the state of knowledge and applicability of AR in Orthopedic surgery. A systematic search of the following three databases was performed: “PubMed”, “Cochrane Library” and “Web of Science”. The systematic review followed the Preferred Reporting Items on Systematic Reviews and Meta-analysis (PRISMA) guidelines and it has been published and registered in the international prospective register of systematic reviews (PROSPERO).

**Results:**

31 studies and reports are included and classified into the following categories: *Instrument / Implant Placement, Osteotomies, Tumor Surgery, Trauma,* and *Surgical Training and Education*. Quality assessment could be performed in 18 studies. Among the clinical studies, there were six case series with an average score of 90% and one case report, which scored 81% according to the Joanna Briggs Institute Critical Appraisal Checklist (JBI CAC). The 11 cadaveric studies scored 81% according to the QUACS scale (Quality Appraisal for Cadaveric Studies).

**Conclusion:**

This manuscript provides 1) a summary of the current state of knowledge and research of Augmented Reality in orthopedic surgery presented in the literature, and 2) a discussion by the authors presenting the key remarks required for seamless integration of Augmented Reality in the future surgical practice.

**Trial registration:**

PROSPERO registration number: CRD42019128569.

## Background

Computer technologies play a crucial role in orthopedic surgery. Up to recently, surgical planning was routinely done manually on fluoroscopy images. Today, this is replaced by advanced planning software incorporating multi-modal and patient-specific medical data. In addition to pre-operative planning, the surgeon is increasingly supported by computers intra-operatively. As an example, during arthroplasty procedures, computer-aided techniques showed to be superior compared to the conventional implantation techniques in regard to both consistency and accuracy [1–6].

Robot solutions are proposed to reduce human error, increase precision, and ensure reproducibility [7–10]. However, they are not yet clinically adopted widely across different disciplines. Current drawbacks of robotic solutions in surgery include their minimal adaptive intellectual and haptic behavior, limitations in integrative interpretation and action in complex situations, ill-posed registration to the patient, complex setup, invasive fiducial implantation, and workflow disruption [9].

While robotic technologies mainly aim at supporting surgeons with precise and planned mechanical actions, technologies such as Augmented Reality (AR) increase the ability of the surgeon by intuitive augmentation of medical information. AR refers to the real world augmented with virtual information, as opposed to Virtual Reality (VR), in which the user is confronted with a completely virtual setting [11, 12]. The user’s view is augmented either via monitor-based display system, optical see-through system or video see-through system [13]. With recent commercial products such as Google Glass (Google Inc., Mountain View, California, USA) and Microsoft HoloLens (Microsoft, Redmond, WA), optical see-through systems have gained broad availability. Such, „head-mounted-displays “(HMD) allow a high degree of flexibility by enabling the user to visualize virtual content that is directly overlaid onto the present reality.

An important component of AR is the underlying tracking-system. Tracking is essential when placing virtual objects into the real world in correct relations and positions. Most systems are based on external markers, where a particular pattern in the real world is tracked as a reference [14]. Visual markers are widely used for this purpose, where unique and high contrast patterns are detected by optical cameras [15]. On the other hand, modern systems act independently from such predefined patterns and are referred to as marker-less systems. The marker-less tracking technology is enabled by using several Inertial Measurement Unit (IMU), Red-Green-Blue (RGB) and Infrared sensors on the HMD which allow creating a spatial map of the room and performing real-time inside-out tracking and localization with respect to the environment. Therefore, they are capable of orienting themselves on already present objects, without additional markers [16].

In interventional medicine, AR is already introduced in several specialties, namely, neuro- [17] and visceral-surgeries [18, 19]. Particularly, there are growing numbers of reports on the applications of AR in the field of orthopedic surgery, which are the focus of this systematic review article. The growing interest for AR in orthopedics and trauma is not surprising, since the surgical procedures in orthopedic surgery frequently use 1) visual data such as medical images acquired both pre- and intra-operatively and 2) often include mechanical steps such as screw or implant insertions, osteotomies and correction of deformities that can be visualizing the rigid relations in AR environments. Hence, such technical tasks seem predisposed to applications of AR. In this article, we aim at providing a systematic review of the current state of knowledge and the applicability of AR in orthopedic surgery.

## Methods

### Search design

A systematic search of the following three databases “PubMed”, “Cochrane Library” and “Web of Science” was performed. For this purpose, all studies written in English or German from inception until 1st of March 2019 were included in the search. Combinations of the following keywords were used: *[Orthopedic]*, *[Orthopaedic], [Trauma]* with the terms *[Augmented Reality]* or *[Image Overlay]*.

First, a blinded and independent process of selection based on title and abstract was made by two authors (LJ and OA). Next, a thorough selection of eligible studies was performed by analyzing full texts. Reasons for exclusion were noted. The current systematic review followed the Preferred Reporting Items on Systematic Reviews and Meta-analysis (PRISMA) guidelines [20]. The protocol of this systematic review has been published and registered in the international prospective register of systematic reviews (PROSPERO) under the registration number: CRD42019128569 [21].

### Selection process

Inclusion criteria were: (1) studies in English or German language; (2) minimum level V of Evidence using Oxford Centre for Evidence-Based Medicine 2011 Levels of Evidence [22]; (3) AR was used in musculoskeletal surgery, and (4) applicability was reported.

Exclusion criteria were (1) review articles or oral presentations; (2) non-English/German articles; (3) articles lacking an available full-text; (4) AR was used outside of musculoskeletal surgery. An eligibility screening using titles and abstracts was first performed with subsequent full-text review. Any differences were discussed until a general consensus between all authors was achieved. Finally, based on the subject area where the study was applied, studies were further sub-grouped.

### Data extraction and quality assessment

The quality was evaluated using the Cochrane Risk of Bias assessment tool [23]. The data extraction consisted of: author and year of study, AR display type, scientific area of applicability, and the main findings. The quality of all the studies including real patients were then assessed using the Joanna Briggs Institute Critical Appraisal Checklist (JBI CAC) [24]. A scoring system was then used per study such as studies that answered yes to a question from the checklist scored 2, not clear scored 1 and not scored 0.

Furthermore, the quality of the cadaveric studies was performed using the QUACS scale (Quality Appraisal for Cadaveric Studies) [25]. Each score was then converted into a percentage to harmonize the scoring system.

## Results

### Systematic search

**Fig. 1:**
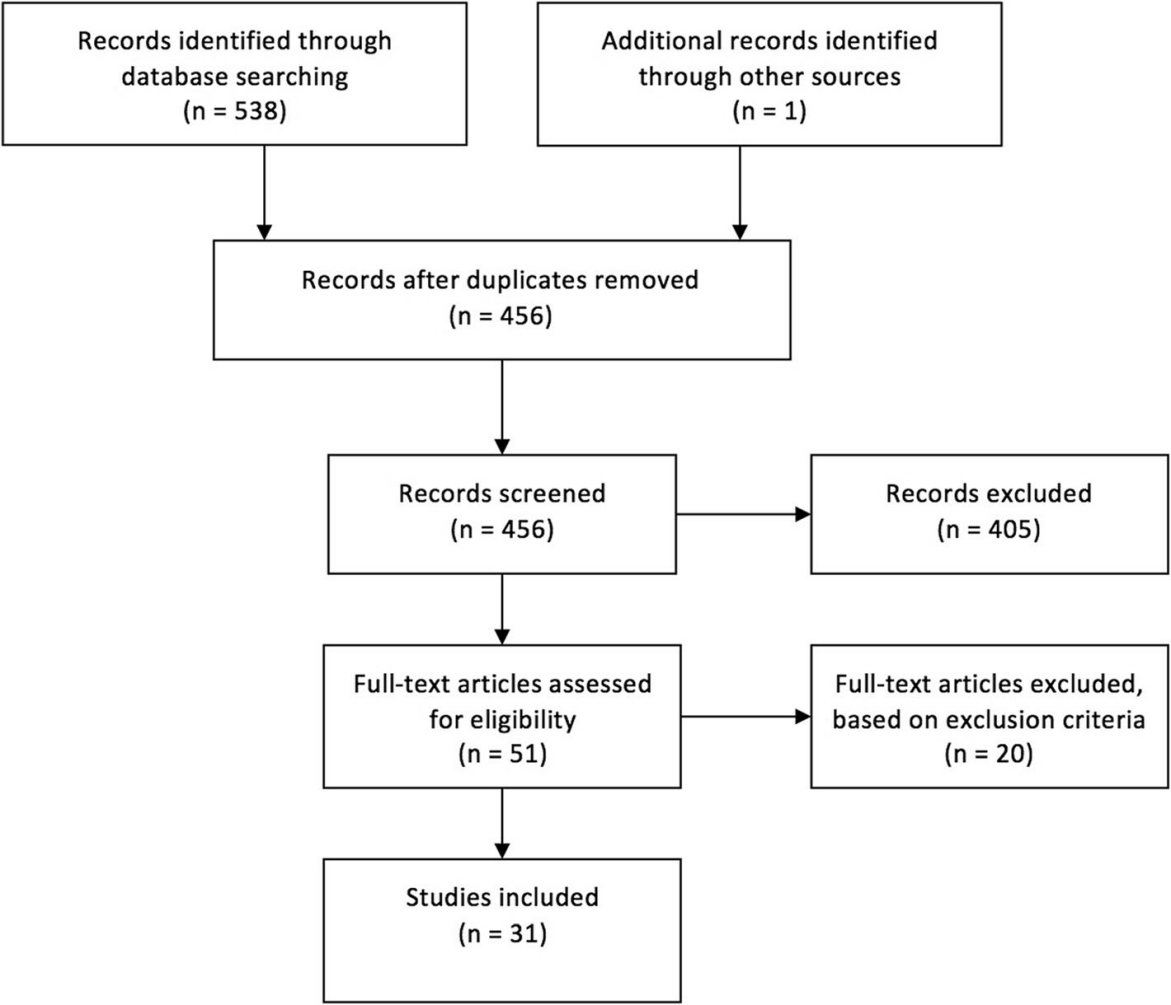
Flowchart of the systematic research in “PubMed” and “Cochrane Libraray”

Based on the above-mentioned predefined search terms and exclusion criteria, 31 studies were selected for final analysis (Fig. 1). The results are summarized in Table 1. During categorization, the included studies have been sub-grouped into the following categories: “*Instrument / Implant Placement*” (20 Studies), “*Osteotomies*” (1 Study), “*Tumor Surgery*” (3 Studies), “*Trauma*” (3 Studies), and “*Surgical Training and Education*” (4 Studies).

**Table 1:** Summary of the included studies

Author	Title	Year	Category	Display-Type	Applicability
Wu JR et al	Real-time advanced spinal surgery via visible patient model and augmented reality system.	2014	Placement	Projector	Yes
Abe Y et al	A novel 3D guidance system using augmented reality for percutaneous vertebroplasty: technical note.	2013	Placement	HMD	Yes
Navab N et al	Camera augmented mobile C-arm (CAMC): calibration, accuracy study, and clinical applications.	2010	Placement	Monitor	Yes
Heining SM et al	Pedicle screw placement under video-augmented flouroscopic control: first clinical application in a cadaver study	2006	Placement	Monitor	Yes
Elmi-Terander A et al	Feasibility and Accuracy of Thoracolumbar Minimally Invasive Pedicle Screw Placement With Augmented Reality Navigation Technology	2018	Placement	Monitor	Yes
Elmi-Terander A et al	Pedicle Screw Placement Using Augmented Reality Surgical Navigation With Intraoperative 3D Imaging: A First In-Human Prospective Cohort Study	2019	Placement	Monitor	Yes
Ma L et al	Augmented reality surgical navigation with ultrasound-assisted registration for pedicle screw placement: a pilot study	2017	Placement	Projector	Yes
Gibby JT et al	Head-mounted display augmented reality to guide pedicle screw placement utilizing computed tomography	2019	Placement	HMD	Yes
U-Thainual P et al	MR image overlay guidance: system evaluation for preclinical use	2013	Placement	Monitor	Yes
Fischer GS et al	MRI image overlay: application to arthrography needle insertion	2007	Placement	Monitor	Yes
Fichtinger G et al	Image overlay guidance for needle insertion in CT scanner	2005	Placement	Monitor	Yes
Fischer M et al	Preclinical usability study of multiple augmented reality concepts for K-wire placement	2016	Placement	Monitor	Yes
Andress S et al	On-the-fly augmented reality for orthopedic surgery using a multimodal fiducial	2018	Placement	HMD	Yes
Befrui N et al	3D augmented reality visualization for navigated osteosynthesis of pelvic fractures	2018	Placement	Monitor	Yes
Londei R et al	Intra-operative augmented reality in distal locking	2015	Placement	Monitor	Yes
Ma L et al	Three-dimensional augmented reality surgical navigation with hybrid optical and electromagnetic tracking for distal intramedullary nail interlocking	2018	Placement	Projector	Yes
Wang H et al	Precision insertion of percutaneous sacroiliac screws using a novel augmented reality-based navigation system: a pilot study	2016	Placement	HMD	Yes
Fotouhi J et al	Plan in 2-D, execute in 3-D: an augmented reality solution for cup placement in total hip arthroplasty	2018	Placement	Monitor	Yes
Ogawa H et al	A Pilot Study of Augmented Reality Technology Applied to the Acetabular Cup Placement During Total Hip Arthroplasty	2018	Placement	Monitor	Yes
Liu H et al	Augmented Reality Based Navigation for Computer Assisted Hip Resurfacing: A Proof of Concept Study	2018	Placement	HMD	Yes
Fallavollita P et al	An augmented reality C-arm for intraoperative assessment of the mechanical axis: a preclinical study	2016	Osteotomies	Monitor	Yes
Cho HS et al	Augmented reality in bone tumour resection: An experimental study	2017	Tumor Surgery	Monitor	Yes
Cho HS et al	Can Augmented Reality Be Helpful in Pelvic Bone Cancer Surgery? An In Vitro Study	2018	Tumor Surgery	Monitor	Yes
Gavaghan K et al	Evaluation of a portable image overlay projector for the visualisation of surgical navigation data: phantom studies	2012	Tumor Surgery	Projector	Yes
Shen F et al	Augmented reality patient-specific reconstruction plate design for pelvic and acetabular fracture surgery	2013	Trauma	Monitor	Yes
Van Duren BH et al	Augmented reality fluoroscopy simulation of the guide-wire insertion in DHS surgery: A proof of concept study	2018	Trauma	Monitor	Yes
Hiranaka T et al	Augmented reality: The use of the PicoLinker smart glasses improves wire insertion under fluoroscopy	2017	Trauma	HMD	Yes
Yeo CT et al	The effect of augmented reality training on percutaneous needle placement in spinal facet joint injections	2011	Training / Education	Monitor	Yes
Ponce B et al	Emerging technology in surgical education: combining real-time augmented reality and wearable computing devices	2014	Training / Education	HMD	Yes
Ponce B et al	Telementoring: use of augmented reality in orthopaedic education: AAOS exhibit selection	2014	Training / Education	Monitor	Yes
Condino S et al	How to Build a Patient-Specific Hybrid Simulator for Orthopaedic Open Surgery: Benefits and Limits of Mixed-Reality Using the Microsoft HoloLens	2018	Training / Education	HMD	Yes

### Quality assessment

The quality assessment process could only be performed in 18 (58%) out of 31 studies, where either human study populations or cadaveric subjects were evaluated. As such, 7 studies (39%) included patients [26–32], and 11 (61%) described cadaveric results [32–42].

Among the clinical studies, there were six case series [26–29, 31, 32] (level IV of Evidence) with an average score 90% (range, 60–100%) according to JBI CAC [24] and one case report [30] (level V of Evidence) which scored 81% (Additional file 1).

The cadaveric studies also scored high based on QUACS scale [25] with an average of 81%; however with a more significant heterogeneity (range 46–100%) (Additional file 2).

## Discussion

The main finding of this systematic review is that AR has the potential to be a timesaving, risk and radiation reducing, and accuracy enhancing technology in orthopedic surgery. AR solutions seem to be well applicable in different fields of orthopedic surgery as highlighted hereinafter.

### Instrument / implant placement

The orthopedic surgeon often relies on his three-dimensional (3D) orientation to place instruments or implants. Intraoperative fluoroscopy provides two-dimensional (2D) information. Therefore, the surgeon has to perform the mental task of mapping the 2D radiographs to the 3D anatomy. AR solutions can potentially reduce the dependence of the outcome on the surgeon’s parameters by providing preoperative planning in the field of view of the surgeon, or even showing correct trajectories for placing implants with overlays. Jing-Ren Wu et al. [32] used a camera-projector AR system to project the spinal bony anatomy on the back of a patient with entry points for vertebroplasty, based on preoperative CT data. For registration, markers were attached to the patient skin and were tracked by the camera. First trials were with a dummy and animal-cadavers. Afterwards, the system was brought to the OR (operating room), and vertebroplasty was performed conventionally but with the additional assistance of AR. Time-saving for entry point identification by 70% was reported. One major limitation was the unreliable registration, in case that the patients’ posture changed between CT and surgery.

**Fig. 2:**
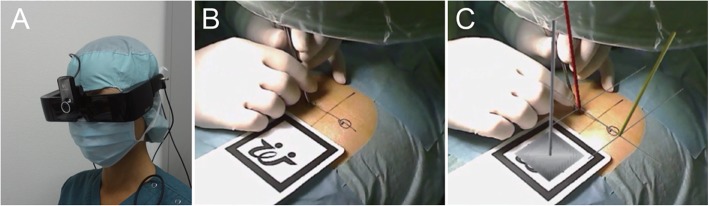
Reprinted by permission from AANS: Journal of Neurosurgery Spine, A novel 3D guidance system using augmented reality for percutaneous vertebroplasty: technical note, Yuichiro Abe, Shigenobu Sato, Koji Kato et al., Copyright 2013. **a**) HMD with camera. **b**) A raw image is captured by the camera. **c**) Actual view of the surgeon. The software creates an augmented view and indicates the ideal insertion point and needle trajectory

Yuichiro Abe et al. [26] simulated needle-insertion into vertebral bodies in a phantom-study. Point and angles of insertion were identified on patients’ preoperative CT scans. During the procedure, the surgeon wore a video see-through HMD (Moverio, Epson) with a webcam. The visual information was observed by the webcam and transmitted to a computer for processing. Registration between the patient and the CT required several manual steps and involved using a few fluoroscopy images. After estimating the spatial relations between the preoperative planning and the patient, the desired trajectories were streamed to the HMD and overlaid on the patient (Fig. 2). Postinterventional CT was used to calculate deviation with respect to the planned trajectory. Significantly higher precision was reported compared to the conventional approach. Following the phantom-trials, they validated their system in five clinical cases and reported successful assistance to the performing surgeon.

Navab N. et al. [41] focused on AR-supported vertebroplasty with a system consisting of a mobile C-arm and a video camera attached near the X-ray source, so-called Camera augmented mobile C-arm (CAMC). This system was designed using a double mirror construction, allowing the origin of the optical and X-ray cameras to virtually coincide. A major advantage of this design was that C-arm fluoroscopy images and video camera frames from the surgical site were fused without the need to warp the images. This system was self-contained and did not require any external navigation system. One goal of their solution was to perform vertebroplasty with one initial X-ray image, which was overlaid onto the video camera image. Five simulated vertebroplasties in a spine model were reported. Maximum of three X-rays were required, which is close to the goal of one. Three of these five procedures showed perfectly positioned needles, and two showed a medial perforation. The main reason for these perforations was reported as undetected motion of the spine. As a consequence, they implemented markers to detect displacement automatically. In the same work, they performed interlocking of intramedullary nails as well as pedicel screw placements in cadavers. The two experiments were conducted successfully, and the procedure required less radiation and time compared to the standard C-arm technique. For the interlocking of intramedullary nails and pedicel screw placement surgeons required two and three X-rays images, respectively. In an earlier study, Heining S. M. et al. [39] also investigated pedicle screw placement using the CAMC system. In two cadaver studies, in different levels of the lumbar and thoracic spine, all needle insertions were possible.

The idea of the CAMC system was further investigated for thoracolumbar pedicle screw placement using an AR capable imaging system [43]. During their preclinical study [35], the surgical table was connected to the C-arm system (AlluraClarity FD20, Philips Healthcare, Best, The Netherlands) whereby the flat detector consisted of four optical cameras. Videos from the cameras were coregistered with the coordinate system of the C-arm. Performing a 3D Cone Beam CT acquisition, screw insertion paths were displayed. Overall 66 Jamshidi needles were placed in two cadavers, and 18 cannulated pedicle screws were placed in one cadaver. Mean error between Jamshidi needles and the planned path was 0.9° ± 0.8°. During this preclinical study, no screw was misplaced outside the pedicle, however, two screws breached, giving an overall accuracy of 89% for screw placement. In a following prospective observational study, Elmi-Terander A. et al. [27] performed 253 lumbosacral and thoracic pedicle screw placements in 20 patients, where they showed an overall accuracy of 94.1%, without any screw severely misplaced.

Ma L. et al. [40] investigated pedicle screw placement by using an ultrasound-assisted registration method. Ultrasound was used to register preoperative CT data with the patient, and surgical navigation was overlaid by an integral videography approach. After agar phantom experiments, sheep cadaver experiments were performed. The mean targeting errors were reported as 3.35 mm and 3.79 mm, respectively. The main advantage of their system was that no repeated radiation was needed due to the use of ultrasound registration.

Another study by Gibby J. T. et al. [44] also investigated pedicle screw placement while using Microsoft HoloLens (Microsoft, Redmond, WA). In a lumbar saw bone model, they placed 36 needles, representing the pedicle screws. Using preinterventional CT data, needle trajectory was estimated and superimposed into the surgeon’s view, Postinterventional CT indicated that 97% of the needles were placed within the pedicle. Calculation with pedicle screws of a diameter up to 7 mm still demonstrated that 86% of screws were placed completely inside the pedicle.

**Fig. 3:**
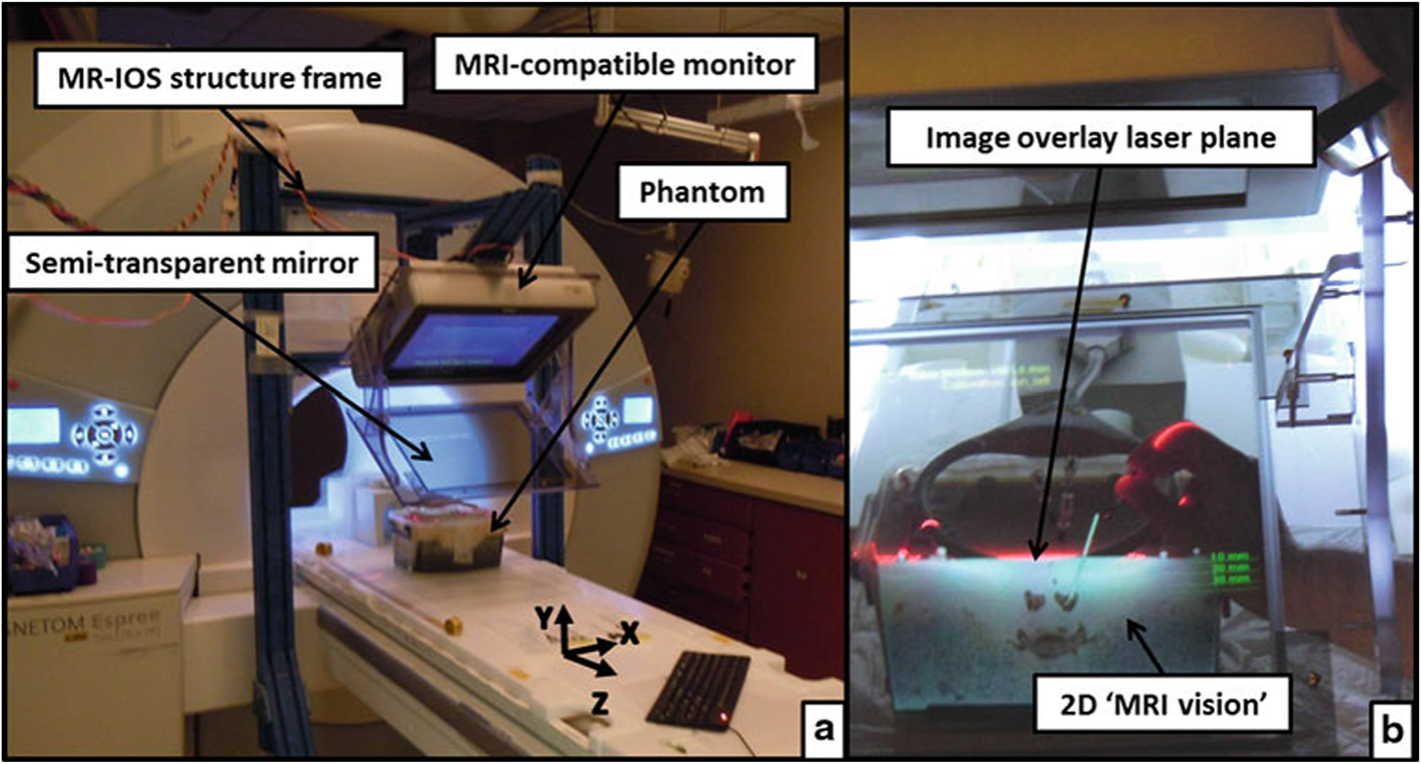
Reprinted by permission from Springer Nature: Springer, International Journal of Computer Assisted Radiology and Surgery, MR image overlay guidance: system evaluation for preclinical use, Paweena U-Thainual, Jan Fritz, Choladawan Moonjaita et al., Copyright 2012. **a**) Visualized is the MR-IOS. **b**) The surgeon looks through the semi-transparent mirror that is augmented with the correct insertion path

U-Thainual P. et al. [45] suggested an AR-based technique for MRI-guided musculoskeletal interventions. The proposed Magnetic Resonance Image Overlay System (MR-IOS) provided an MRI vision for the operator and was used for needle insertions on a spine phantom. Main hardware components included a transverse plane laser, an MRI compatible monitor, and a semi-transparent mirror (Fig. 3). Onto this mirror, the MR image and the desired insertion path were jointly projected. This system was mounted in the mouth of an MRI scanner that provided 2D transverse slices. The alignment between the virtual medical images in the mirror and the patient was achieved by manually rotating and translating the virtual image plane until the anatomical landmarks on the patient and image coincide. 40 novice operators, 20 using the freehand technique and 20 using MR-IOS-guided technique, performed needle insertion. The overlay-group reached significantly better success rates in correct needle placement (overlay-group 80,95% vs. freehand 35,0%) and produced less tissue damage. Procedure time was not significantly different. The authors reported an inherent problem of their proposed mirror display as the increase of refraction error when the scene was observed from oblique angles. Another study from Fischer G.S. et al. [38] also investigated the usefulness of MR-IOS in performing arthrography in porcine and humans’ shoulder and hip joints. In their trial, every needle insertion was successful in the first attempt. A similar construct was built and evaluated by Fichtinger G. et al. [37], where instead of MRI, CT data was used as the baseline. They successfully performed spinal nerve blocks, facet joint injections, shoulder and hip arthrographies, and needle insertions for musculoskeletal biopsy in cadaver experiments. Limitations included complex calibration phase, interference of the room light with the overlay, and the parallax effect.

Fischer M. et al. [46] compared minimally invasive placement of K-wire into a fractured superior pubic ramus with three different systems: I) conventional C-arm, II) C-arm augmented by a video camera, as described by Navab N. et al. in [41], and III) a cone-beam CT (CBCT) enabled C-arm augmented by a Red-Green-Blue Depth (RGBD) camera. System III allowed simultaneous visualization from multiple arbitrary views, as opposed to just 2D visualization in the others. Rendering and visualization of the AR scene from multiple perspectives allowed the surgeons to align their tools with the anatomy from several desired perspectives, simultaneously. By first acquiring a CBCT scan of a calibration phantom, the co-registration between the RGBD and CBCT coordinate frames were performed. While acquiring projection images for this CBCT scan, the surface of the phantom was also reconstructed using data from the RGBD camera. The rigid body transformation expressing the geometric relation of the C-arm and the RGBD camera was estimated using the CBCT and the surface reconstruction data. This calibration will remain valid as long as the camera is not displaced on the C-arm. They performed 21 K-wire placements. The AR system with the RGBD camera yielded the highest benefit with regard to the duration of the procedure and radiation exposure. Using a standardized questionnaire, the authors measured the surgical task load and reported a significant reduction. A Limitation was that the augmentation becomes invalid when the C-arm was rotated to a different angle.

An on-the-fly surgical support system for percutaneous image-guided orthopedic procedures in un-prepared OR environments is proposed by Andress S. et al. [47]. The hardware components of this system include a C-arm imaging device, a Microsoft HoloLens (Microsoft, Albuquerque, New Mexico, USA) optical-see-through HMD, and a hybrid fiducial. The relation between the X-ray source and HMD is recovered every time the multi-modal fiducial is introduced into the field of view of both C-arm X-ray and the HMD. Thereafter, annotations on X-ray images are visualized as 3D virtual lines that provide surgical guidance. In a pre-clinical feasibility study, medical experts placed K-wires into a semi-anthropomorphic femur phantom using the suggested on-the-fly AR system. The average error was reported as 5.20 mm.

Using the RGBD augmented CBCT system [46], Befrui N. et al. [48] performed K-wire placement in a long bone phantom and a superior pubic ramus phantom. For control, K-wire placement was also performed using conventional C-arm fluoroscopy alone. Procedure time when using AR navigation was significantly reduced from 9.9 min to 4.1 min, respectively, from 10.9 min to 5.5 min. Radiation dose was also relevantly reduced in both procedures when using the AR approach. Regarding the placement accuracy, no significant difference was observed between the conventional and the AR approach.

Londei R. et al. [49] used the camera augmented C-arm proposed by Navab N et al. [41] and performed studies on distal locking of intramedullary (IM) nails, a procedure which requires a large number of C-arm fluoroscopic images. In this work, “down-the-beam” view of the IM nail was achieved by first acquiring an X-ray image of the nail.

**Fig. 4:**
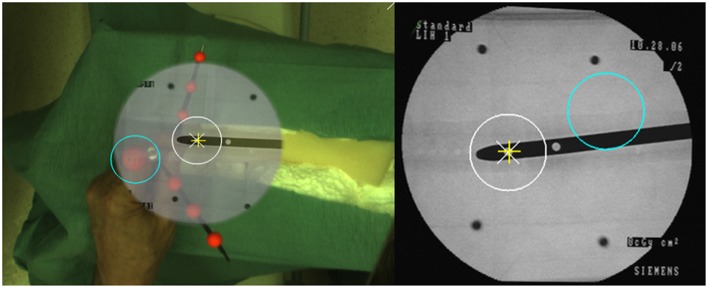
Visualized are the perfect circles for distal locking

They registered the information of this image with the 3D computer-aided design (CAD) model of the IM nail to estimate the C-arm pose and subsequently predict a second view that produces the “perfect circle” view of the holes on the IM nail (Fig. 4). Therefore, the authors were able to conduct intramedullary nailing and distal locking with only two X-ray images, on average. To track the drill, a cannula with chained cross-ratios was placed on the surgical drill. The markers on this cannula were tracked by a video camera on the C-arm, and the position of the drill tip was estimated with respect to the patient. This system was used in 70 procedures performed by four participants (2 experts, 1 resident, and 1 medical student) on a dry bone model. Results indicated a success rate of 93%. The average time was reported as 100 s only.

Ma L. et al. [50] also investigated locking of IM nails by using an AR navigation system with hybrid optical and electromagnetic tracking. 3D image overlays were provided using an integral videography approach. During their pre-clinical study, 18 drills were successfully placed in five tibia models and a leg phantom.

Wang H. et al. [42] performed AR-based navigation for percutaneous placement of sacroiliac screws in six cadavers. Preoperatively, the authors acquired CT scans of each pelvis and segmented the bone and vessels from other tissue in the CT data. Given the CT images, ideal entry points and trajectories of the percutaneous screws were calculated. This system was materialized by registering the preoperative data and the planned trajectories to the cadaver and projecting the surgical plan as a cylinder onto a HMD display. Their hardware included an optical see-through HMD (nVisor ST60, NVIS, USA) and an external optical navigation system that tracked reflective fiducials on the HMD, surgical drill, and the cadaver. During their experiments, the surgeon was able to visualize the estimated entry point and match the angle of screw insertion with the projected cylinder. All screws were implanted successfully with only a few millimeters of aberration from planning. No bony perforation was reported. Major limitations were complex setup due to the use of external navigation system, out-side-in tracking of different components, and the bulky and tethered setup.

**Fig. 5:**
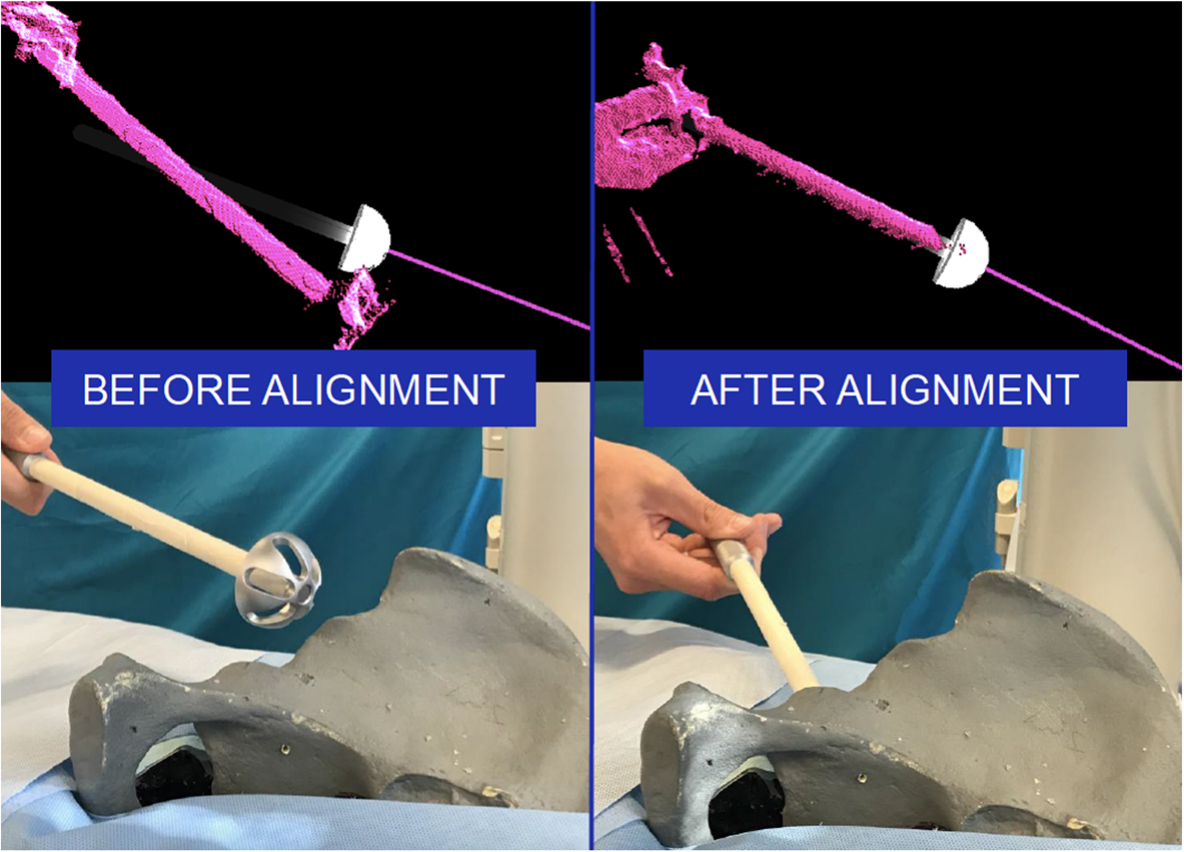
The surgeon sees multiple virtual perspectives of the surgical site and moves the impactor until it completely overlaps with the virtual planning

Fotouhi J. et al. [51] proposed an intra-operative AR guidance system for acetabular cup placement in total hip arthroplasty. Using two intraoperative acquired C-arm X-ray images, the cup position is planned. Next, an RGBD augmented C-arm system was used to fuse the visual information of the surgical scene with the desired planning of the cup and impactor (Fig. 5). A pre-clinical feasibility study was performed to evaluate acetabular cup planning using stereo X-ray imaging. Four orthopedic residents used the planning software to place the cup on simulated X-ray images. The results indicated that the average error in abduction and anteversion compared to classic direct anterior approach improved by 6.5° and 1.8°, respectively.

Another study by Ogawa H. et al. [28] investigated cup placement in total hip arthroplasty measured by an AR approach. In 56 total hip arthroplasties, the orientation of the acetabular cup (anteversion and inclination) were measured either using an AR smartphone or a goniometer. Three months postoperatively, cup anteversion and inclination were measured in CT. No statistically significant difference between AR and the goniometer was observed concerning the inclination, whereas anteversion was significantly more accurate using the AR approach (*p* < 0.0001).

Liu H. et al. [52] performed a proof of concept study about AR-based navigation in hip resurfacing. Using a robotic system [53] and the Microsoft HoloLens (Microsoft, Albuquerque, New Mexico, USA) they suggested a system to support the drilling of a hole along the axis of a femoral neck. Comparing the postinterventional drill orientation with the preinterventional plan in a phantom study yielded a mean error of approximately 2 mm and 2°.

### Osteotomies

In high tibial osteotomy, knowledge about the mechanical axis is essential. Based on the work of Wang L. et al. [54], Fallavollita P. et al. [36] published a study on intraoperative assessment of the mechanical axis of the lower limb using AR navigation. Using the conventional fluoroscopy-based approach, a large number of X-ray images were required. Using the RGB camera setup on the C-arm, with only three X-ray images - one from the hip, one from the knee and one from the ankle - a parallax-free panoramic image of the lower limb was created. Their innovative parallax-free solution required a joint rotation of the C-arm scanner around the origin of the X-ray source as well as the translation of the surgical bed. These two motions were applied such that the overall translation around the X-ray origin vanished, and therefore, the parallax effect was eliminated. Using this intraoperatively acquired non-overlapping panoramic image, the authors measured the mechanical axis and the amount of misalignment that required correction for high tibial osteotomy. Procedures were carried out in 25 human cadaver legs. To evaluate the usefulness of the proposed AR solution, the mechanical axis, and its respective deviation were also measured in the ground-truth CT scan. The AR system proved to be an accurate and low radiation technique.

### Tumor surgery

Information about the 3D expansion of the tumor is crucial for a proper resection. Cho H. S. et al. addressed this problem by incorporating AR support [33, 34]. The first study faced the subject of tumor resection in pig femurs. Multiple visual markers were attached to the subject, which was tracked by the integrated camera on a tablet PC. In this overlay, a cylindrical virtual template represented the tumor. The virtual implant was then superimposed on the patient using the tablet PC. Tumors were simulated in 123 pig femurs. Resection was performed in 82 femurs using the AR-based approach and in 41 femurs using the conventional approach. The probability of reaching the safe margin of 10 mm with a tolerance of 3 mm was 90.2% in the AR approach versus 70.7% in the conventional technique. The second study of Cho H. S. et al. faced the subject of tumor resection in pig pelvis models. 36 pig pelvis models were prepared with simulated bone tumors in the acetabular dome, using bone cement. 18 tumors were resected using the same AR-based navigation method, enabled by the tablet PC. The remaining 18 pelves were operated using the conventional approach. Resection was planned with a 1 cm safety margin. All AR-assisted resections yielded errors < 6 mm, whereas in the conventional group merely 78% had resection errors < 6 mm.

Gavaghan K. et al. [55] also investigated the applications of AR in orthopedic tumor surgery. The authors used a hand-held RGB laser projector (PicoPMicrovision, US) that projected an image onto an intraoperative situs. Their proposed system eliminated the need for in-direct visualization and enabled direct visualization in the surgical site. Registration between the patient and the CT data was achieved by using a landmark-based registration approach. Bone tumors were simulated via 3D printed proximal tibias. The projector showed the tumor directly on the model, including a previously defined resection margin. Due to the loss of depth perception, only 2D resection lines were effectively visualized compared to 3D resection planes. The resection of the tumor, as it’s needed in such cases, was not performed in this trial.

### Trauma

Shen F. et al. [31] developed an AR implant design system for preoperative creation of osteosynthesis plates in unilateral pelvic and acetabular fractures. The proposed solution comprised two sub-systems: I) a virtual fracture reduction system in which a repaired model of the fractured pelvis was constructed and an ideal curve indicating the implant model was identified, II) an AR templating environment to manipulate and bend the implant according to the planned trajectory. This AR system consisted of an external monitor and a high definition (HD) webcam. The suggested technology enabled the surgeon to visualize the physical implant as well as the augmentation of the virtual model simultaneously and use the AR environment to create the desired implant model. The reduction was performed on six fractured pelvises. Preoperative CT was acquired, and fracture reduction was performed on the computer. For each case, virtual osteosynthesis plates were first drawn in ideal locations. This information was used together with the aid of the AR system to bend the osteosynthesis plates. This allowed pre-bent osteosynthesis plates for ideal fracture reduction. The authors claimed that the intraoperative implant bending could be eliminated using their approach; therefore surgical time and invasiveness could be minimized.

Van Duren B. H. et al. [56] investigated an AR fluoroscopy simulation for guide-wire insertion in dynamic hip screws. Their system included cameras that were orthogonally viewing the operative site and tracking the marked guide-wires. Postinterventional, the tip-apex distance (TAD) between the guide-wire and the femoral head was measured with a mean square error of 4.2 mm. An increase of accuracy with the number of iterations was observed with an error of 2 mm.

Another study by Hiranaka T. et al. [57] also evaluated guide-wire insertion into five artificial femoral heads by the use of AR navigation. They made use of the PicoLinker wearable glasses (Westunits Co., Ltd., Osaka, Japan) that was connected to the fluoroscopic monitor such that the surgeon was able to observe the fluoroscopic video through the PicoLinker glasses. Wire insertion was performed ten times using the AR approach and ten times using the fluoroscopy alone. Postoperatively TAD was measured significantly smaller in the AR approach than using the conventional approach (2.6 mm respectively 4.1 mm, *p* = 0.02). Likewise, both radiation time and total insertion time were significantly shorter by using the AR approach.

### Surgical training and education

Yeo C. T. et al. [58] suggested the employment of AR for spine surgery training. In their work, the AR simulation display was designed using a semi-reflective glass, where a slice of the CT, as well as the trajectory of the needle, were augmented onto the trainee’s view. An electromagnetic tracker was used to estimate needle pose in relation to CT. The trajectory was then projected onto the AR display using a laser-guided system. The authors compared two groups: I) the first group received AR supported training, and II) the control group received training for conventional freehand facet joint injections. Later, both groups performed injections in a phantom with the conventional freehand technique. The AR-trained group achieved higher rates of successful placement of injections with less tissue trauma compared to the control group.

Ponce B. et al. [30] introduced an AR-based surgical training system for tele-guided shoulder arthroplasty. This system, so-called virtual interactive presence (VIP), allowed a physically absent surgeon to be virtually present. One video camera in the OR and one at the remote station were first calibrated. Thereafter, both surgeons were able to observe the surgical site concurrently with a common task field. By using Google Glass (Google Inc., Mountain View, California, USA), the second surgeon that was physically absent was able to join their collaborative virtual experience. The remote surgeon could follow the entire procedure and provide real-time feedback. The authors reported several technical issues they encountered, such as battery life of the HMD, poor video quality, limited field of view, and video mismatch due to delay.

In another report, Ponce B. et al. [29] used the VIP technology for rotator cuff and shoulder instability interventions. Six different resident surgeons performed the procedures, and one attending surgeon that was physically located in an adjoining dictation room proctored the procedures using the VIP technology. The attending surgeon was able to see the arthroscopic image at any time and was able to guide the residents.

Condino S. et al. [59] performed a study on how to build a patient-specific hybrid simulator for orthopaedic open surgery. By using a Microsoft HoloLens HMD and patient-specific 3D models, five subjects performed hip arthroplasty. Using their simulator, they reported that the perceived positioning accuracy matched the requirements, and the overall workload was low.

To date, AR is not yet widely adopted in the clinical routine of orthopedic interventions. Most of the identified studies are preclinical and demonstrate proof-of-concept findings. Nevertheless, AR solutions seem to be well suited for different interventions and are applicable to different fields of orthopedic surgery, including the ones that were highlighted in this systematic review paper.

Most of the presented studies require complex registration between the AR system and the patient. These registration techniques often rely on external navigation systems that suffer from line-of-sight issues; therefore limit the free movement of the surgical crew and the imaging device (e.g., C-arm). Often, registration of these landmarks demands high manual interaction to solve the ill-posed registration task, which leads to workflow disruption. The tradeoff between the system accuracy and surgical workflow has to be carefully considered when designing such AR solutions for the operating rooms of the future. Due to the limitations of marker-less tracking, and the complexities of fiducial-based tracking techniques, surgical AR-based systems cannot directly replace the classic navigation systems. Instead, they should be regarded as advanced visualization techniques that can be used to present the medical information optimally to the surgeon.

Visualization of the medical data in an AR environment requires careful consideration regarding the design of realistic perceptual cues. Incorrect perception can lead to geometric ambiguities in identifying the scale of objects, complicate the interaction between real and virtual information, and therefore hamper the adoption of the AR. One important display property to consider in commercially available HMDs is the vergence and accommodation conflict, which can prohibit a sharp rendering of virtual content at arbitrary distances.

Lastly, it is essential to provide user-friendly interfaces. Lack of intuitive design can limit the surgeon in employing the technology for their clinical use. In a human-centered AR system, in addition to the user interface, the user experience plays an important role and has to be aligned with the requirements of the surgeon as the key stakeholder.

Overall, AR has the potential to be a timesaving, risk and radiation reducing, and accuracy enhancing technology in orthopedic surgery. In addition to augmenting the surgeons’ view with useful information, AR appears as well to be a valuable tool in surgical simulation and intraoperative education. With the currently available and expected increase in computational power, it can be expected that AR experiences a geometric increase in applicability in the field of orthopedic surgery. However, with future studies, it will be important to further evaluate the clinical differences of AR in term of cost-reduction and improvements in patient care.

## Conclusion

This manuscript provides 1) a summary of the current state of knowledge and research of Augmented Reality in orthopedic surgery presented in the literature, and 2) a discussion by the authors presenting the key remarks required for seamless integration of Augmented Reality in the future surgical practice.

## Supplementary information

**Additional file 1.** Joanna Briggs Institute Critical Appraisal Tool For Case Report Studies

**Additional file 2.** QUACS (Quality Appraisal for Cadaveric Studies)

## Data Availability

All data generated or analyzed during this study are included in this published article [and its supplementary information files].

## Abbreviations

2D: Two-dimensional
3D: Three-dimensional
AR: Augmented Reality
CAD: Computer-aided design
CAMC: Camera augmented mobile C-arm
CBCT: Cone-beam computed tomography
CT: Computed tomography
HD: High definition
HMD: Head-mounted-displays
IM: Intramedullary
IMU: Inertial Measurement Unit
JBI CAC: Joanna Briggs Institute Critical Appraisal Checklist
MRI: Magnetic resonance imaging
MR-IOS: Magnetic Resonance Image Overlay System
OR: Operating room
PRISMA: Preferred Reporting Items on Systematic Reviews and Meta-analysis
PROSPERO: International prospective register of systematic reviews
QUACS: Quality Appraisal for Cadaveric Studies
RGB: Red-Green-Blue
RGBD: Red-Green-Blue Depth
TAD: Tip-apex distance
VIP: Virtual interactive presence
VR: Virtual Reality
