# Supplementary material for: Applicability of augmented reality in orthopedic surgery – A systematic review
Source: BMC Musculoskelet Disord. 2020 Feb 15;21:103. doi: 10.1186/s12891-020-3110-2 (PMC7023780; doi:10.1186/s12891-020-3110-2)
Supplement: Supplementary file 1 — Additional file 1. Joanna Briggs Institute Critical Appraisal Tool For Case Report Studies [file 12891_2020_3110_MOESM1_ESM.docx]

| JOANNA BRIGGS INSTITUTE CRITICAL APPRAISAL TOOL FOR CASE REPORT STUDIES  Yes = 2 Unclear = 1 No = 0 NA | | | | | | | | | | |
| --- | --- | --- | --- | --- | --- | --- | --- | --- | --- | --- |
| Study | 1. 1. Were patient’s demographic characteristics clearly described? | 1. 2. Was the patient’s history clearly described and presented as a timeline? | 1. 3. Was the current clinical condition of the patient on presentation clearly described? | 1. 4. Were diagnostic tests or assessment methods and the results clearly described? | 1. 5. Was the intervention(s) or treatment procedure(s) clearly described? | 1. 6. Was the post-intervention clinical condition clearly described? | 1. 7. Were adverse events (harms) or unanticipated events identified and described? | 1. 8. Does the case report provide takeaway lessons? | Total score | % |
| Ponce BA et al, 2014 | 1 | 0 | 2 | 2 | 2 | 2 | 2 | 2 | 13/16 | 81% |

| JOANNA BRIGGS INSTITUTE CRITICAL APPRAISAL TOOL FOR CASE SERIES STUDIES  Yes = 2 Unclear = 1 No = 0 NA | | | | | | | | | | | | |
| --- | --- | --- | --- | --- | --- | --- | --- | --- | --- | --- | --- | --- |
| Study | 1. 1. Were there clear criteria for inclusion in the case series? | 1. 2. Was the condition measured in a standard, reliable way for all participants included in the case series? | 1. 3. Were valid methods used for identification of the condition for all participants included in the case series? | 1. 4. Did the case series have consecutive inclusion of participants? | 1. 5. Did the case series have complete inclusion of participants? | 1. 6. Was there clear reporting of the demographics of the participants in the study? | 1. 7. Was there clear reporting of clinical information of the participants? | 1. 8. Were the outcomes or follow up results of cases clearly reported? | 1. 9. Was there clear reporting of the presenting site(s)/clinic(s) demographic information? | 1. 10. Was statistical analysis appropriate? | 1. TOTAL | 1. % |
| Ogawa H et al, 2018 | 2 | 2 | 2 | 2 | 2 | 2 | 2 | 2 | 2 | 2 | 20/20 | 100 |
| Elmi-Terander et al, 2019 | 2 | 2 | 2 | 2 | 2 | 2 | 2 | 2 | 2 | 2 | 20/20 | 100 |
| Shen F et al, 2013 | 2 | 2 | 2 | 2 | 2 | 2 | 2 | 2 | 2 | 2 | 20/20 | 100 |
| Ponce BA et al, 2014 | 1 | 2 | 2 | 2 | 2 | 0 | 0 | 0 | 1 | 2 | 12/20 | 60 |
| Abe Y et al, 2013 | 2 | 2 | 2 | 2 | 2 | 2 | 2 | 2 | 2 | 2 | 20/20 | 100 |
| Wu JR et al, 2014 | 1 | 2 | 1 | 2 | 2 | 2 | 0 | 2 | 2 | 2 | 16/20 | 80 |
