# Supplementary material for: Applicability of augmented reality in orthopedic surgery – A systematic review
Source: BMC Musculoskelet Disord. 2020 Feb 15;21:103. doi: 10.1186/s12891-020-3110-2 (PMC7023780; doi:10.1186/s12891-020-3110-2)
Supplement: Supplementary file 2 — Additional file 2. QUACS (Quality Appraisal for Cadaveric Studies) [file 12891_2020_3110_MOESM2_ESM.docx]

| QUACS (Quality Appraisal for Cadaveric Studies)  Yes = 1 No = 0 | | | | | | | | | | | | |
| --- | --- | --- | --- | --- | --- | --- | --- | --- | --- | --- | --- | --- |
| **Item** | Wu JR et al, 2014 | Navab N et al, 2010 | Fischer GS et al, 2007 | Fichtinger G et al, 2005 | Wang H et al, 2016 | Fallavollita P et al, 2016 | Cho HS et al, 2017 | Cho HS et al, 2018 | Elmi-Terander et al, 2018 | Heining, S et al, 2006 | Ma L et al, 2017 |  |
| Objective stated | 1 | 1 | 1 | 1 | 1 | 1 | 1 | 1 | 1 | 1 | 1 |  |
| Basic information about sample is included | 1 | 1 | 1 | 1 | 1 | 1 | 1 | 1 | 1 | 1 | 1 |  |
| Applied methods are described comprehensibly | 1 | 1 | 1 | 1 | 1 | 1 | 1 | 1 | 1 | 1 | 1 |  |
| Study reports condition of the examined specimens | 0 | 0 | 0 | 0 | 1 | 1 | 0 | 0 | 1 | 0 | 0 |  |
| Education of dissecting researchers is stated | 0 | 0 | 1 | 0 | 1 | 1 | 1 | 1 | 1 | 0 | 0 |  |
| Findings are observed by more than one researcher | 1 | 1 | 1 | 1 | 1 | 1 | 1 | 1 | 1 | 1 | 1 |  |
| Results presented thoroughly and precise | 1 | 1 | 1 | 0 | 1 | 1 | 1 | 1 | 1 | 0 | 1 |  |
| Statistical methods appropriate | 0 | 0 | 0 | 0 | 1 | 1 | 1 | 1 | 1 | 0 | 0 |  |
| Details about consistency of findings are given | 0 | 1 | 1 | 1 | 1 | 1 | 1 | 1 | 1 | 0 | 0 |  |
| Photographs of the observations are included | 1 | 1 | 1 | 1 | 1 | 1 | 1 | 1 | 1 | 1 | 1 |  |
| Study is discussed within the context of the current evidence | 1 | 1 | 1 | 1 | 1 | 1 | 1 | 1 | 1 | 0 | 1 |  |
| Clinical implications of the results are discussed | 1 | 1 | 1 | 1 | 1 | 1 | 1 | 1 | 1 | 1 | 1 |  |
| Limitations of the study are addressed | 1 | 1 | 1 | 1 | 1 | 1 | 1 | 1 | 1 | 0 | 1 |  |
| Totals (%) | 9/13 (69%) | 10/13 (77%) | 11/13 (85%) | 9/13 (69%) | 13/13 (100%) | 13/13 (100%) | 12/13 (92%) | 12/13 (92%) | 13/13 (100%) | 6/13 (46%) | 9/13 (69%) |  |
